# Nano‐Infrared Imaging and Spectroscopy of Animal Cells in Liquid Environment

**DOI:** 10.1002/smll.202507097

**Published:** 2025-10-14

**Authors:** Alexander Veber, Cecilia Spedalieri, Janina Kneipp

**Affiliations:** ^1^ Department of Chemistry Humboldt‐Universität zu Berlin Brook‐Taylor‐Str. 2 12489 Berlin Germany; ^2^ Institute for Electronic Structure Dynamics Helmholtz‐Zentrum Berlin für Materialien und Energie GmbH Albert‐Einstein‐Str. 15 12489 Berlin Germany

**Keywords:** culture medium, fibroblast cell, live cells, nano‐IR, near field, scattering‐type scanning near field optical microscopy (s‐SNOM), SiC membrane, SiN membrane

## Abstract

Infrared nano‐spectroscopy (nano‐IR) using scattering‐type near‐field optical microscopy (s‐SNOM) is becoming an important tool for analyzing vibrational spectra in nanometer‐scale volumes of intact samples. Recent s‐SNOM experiments using ultrathin membranes have enabled nanoscopy of cells and biomolecules in liquid environments. This study reports the use of SiC membranes as a stable, biocompatible interface between cells in their medium and the atomic force microscopy (AFM) probe of the s‐SNOM, ensuring broadband nano‐IR experiments above 1050 cm^−1^. Nano‐IR images and spectra are collected from fibroblast cells grown adherently to confluence on the membranes using a broadband synchrotron IR source, probing the membrane‐adjacent cellular region. Optimized tip‐sample interaction allows to significantly increase the signal‐to‐noise‐ratio in nano‐IR imaging. The alteration of the tapping amplitude and set‐point of the AFM cantilever allows to adjust the probing depth and suggests a nano‐IR tomography approach that uses a single demodulation harmonic of the signal. The nano‐IR spectra of fibroblast cells resemble their far‐field spectra, but reflect a heterogeneity in the composition and structure of proteins, nucleic acids, carbohydrates, and of membrane lipid organization. The results demonstrate nano‐IR probing of complex samples in liquid media and suggest ways to improve efficiency and standardization of existing approaches in vibrational nanoscopy.

## Graphical abstract

The use of SiC membranes as a stable, biocompatible interface between cells in their medium and the scanning probe of an infrared scattering‐type scanning near field optical microscope is shown to yield nano‐IR images and spectra from living fibroblast cells using a broadband synchrotron IR source. New possibilities for adjustments of probing depth for use in nanotomography are proposed.

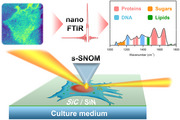

## 1 Introduction

Infrared microspectroscopy has been used for about three decades for the characterization of animal cells and tissues, based on the rich information about the biomolecular composition, structure, and interaction in a sample that is contained in infrared vibrational spectra. With the utilization of scattering‐type near‐field optical microscopes (s‐SNOM) in the infrared range,^[^ ^1^ ^]^ vibrational spectra can be obtained from nanoscale volumes,^[^ ^2^, ^3^ ^]^ in a large variety of materials, with a spatial resolution down to ≈ 10 nm.^[^ ^4^, ^5^ ^]^ As the technique allows to collect unique information about the chemistry of few and individual macromolecules or nano‐ and microdomains in materials, it became a breakthrough in the development of the vibrational nanoscopy field. The availability of light sources for infrared nano‐spectroscopy (nano‐IR), such as quantum cascade lasers and synchrotron infrared beamlines have rapidly progressed, extended the frequency range,^[^ ^6^, ^7^ ^]^ and contributed to deepening of the fundamental understanding of the method and to widening of its application area.

In the field of biomaterials characterization, nano‐IR using an s‐SNOM has been moving from proof‐of‐principle of imaging biological nanoobjects^[^ ^8^, ^9^, ^10^ ^]^ to studies of individual protein molecules and their assemblies,^[^ ^11^, ^12^, ^13^, ^14^ ^]^ and the characterization of structure‐function relationships of complex functional biomaterials such as cell walls in fungi and plants,^[^ ^15^, ^16^ ^]^ or the distribution of drugs in cells.^[^ ^17^ ^]^

For many applications, in particular in studies of functional biological samples, it is crucial to perform the nano‐IR experiment in liquid environment, and at defined osmolarity and pH. As examples, in order to characterize functional proteins, their spectra must be obtained in physiological buffers or in their usual membrane environments. The structure and interaction of biomolecules in cells and cellular compartments change upon additional fixation and drying of the samples. Similarly, the probing of reactions at liquid‐solid interfaces is critical for different branches of research.

Compared to other scanning probe techniques, including tip‐enhanced Raman scattering that is extremely challenging to obtain from liquid biosamples,^[^ ^18^, ^19^ ^]^ nanoscale vibrational probing and imaging of biomolecules by nano‐IR can be attained without direct interaction of the tip with the molecule and in the absence of strong field gradients. Different approaches have been successful in studies by nano‐IR in water, such as the use of a fluid cell with total internal reflection illumination (TIR s‐SNOM, i.e., evanescent‐field IR) in experiments with functional enzymes^[^ ^20^ ^]^ or the acquisition of nano‐IR images of halobacterium purple membranes by use of a silicon solid immersion lens.^[^ ^21^ ^]^ The first approaches to in‐liquid s‐SNOM experiments proposed to cover wet objects by single graphene sheets,^[^ ^22^ ^]^ or encapsulation between two graphene sheets, which, for example, enabled nano‐IR spectroscopy of virus suspensions.^[^ ^23^ ^]^ Nevertheless, the high pressure in such a chamber and compression of samples between two membranes excludes use of such an environment for in situ growth of animal cells and compromise their morphology and integrity. Recently, nano‐IR s‐SNOM was used to collect images and spectra through an ultra‐thin SiN membrane.^[^ ^24^ ^]^ Similar to a graphene sheet, the membrane enables to avoid the direct mechanical contact between the AFM probe and the liquid sample. It was shown that using a simple liquid sample environment with the SiN membrane as a window allows to collect nano‐IR images from a living epithelial cell drop‐casted to it and from bacteria in suspension.^[^ ^24^ ^]^

Here, we report in‐liquid s‐SNOM experiments that make use of a biocompatible and stable ultra‐thin transparent membrane suited for in vivo probing of animal cells. Specifically, using the example of a fibroblast cell line, we show that adherent cells can be grown to confluence directly on the membrane surface, where they remain during the measurement, and where nano‐IR experiments can be conducted both in buffer as well as in protein‐supplemented culture medium.

To enable the acquisition of nano‐IR spectra of the cells in a broad spectral range, we use a synchrotron light source, and robust 30 nm SiC membranes as the main substrate material. SiC is a common material used for the fabrication of ultra‐thin membranes, and its use beneficial in the spectral ranges 1 –3 nm and 10 –20 nm, as well as shown to be promising for infrared spectroscopy applications when compared to SiN.^[^ ^25^, ^26^ ^]^ To estimate the spectral range accessible in in‐liquid IR s‐SNOM we collected broadband nano‐IR spectra of an aqueous phosphate buffer using two types of membranes.

As will be shown, experiments on cells immersed in buffer with varied tip‐sample interaction parameters indicate the possibility to gradually change nano‐spectroscopic probing depth in the sample, without disturbing the adhesion of the cells to the membrane at the nanometer scale. As will be discussed and supported by data of pure protein and DNA molecules, the nano‐IR spectra collected from the living cells reveal differences that give evidence of their structural and compositional heterogeneity that is in agreement with results obtained by other biophysical approaches.

## 2 Results and Discussion

### 2.1 Infrared Optical Properties of SiN and SiC Membranes

**Figure 1:**
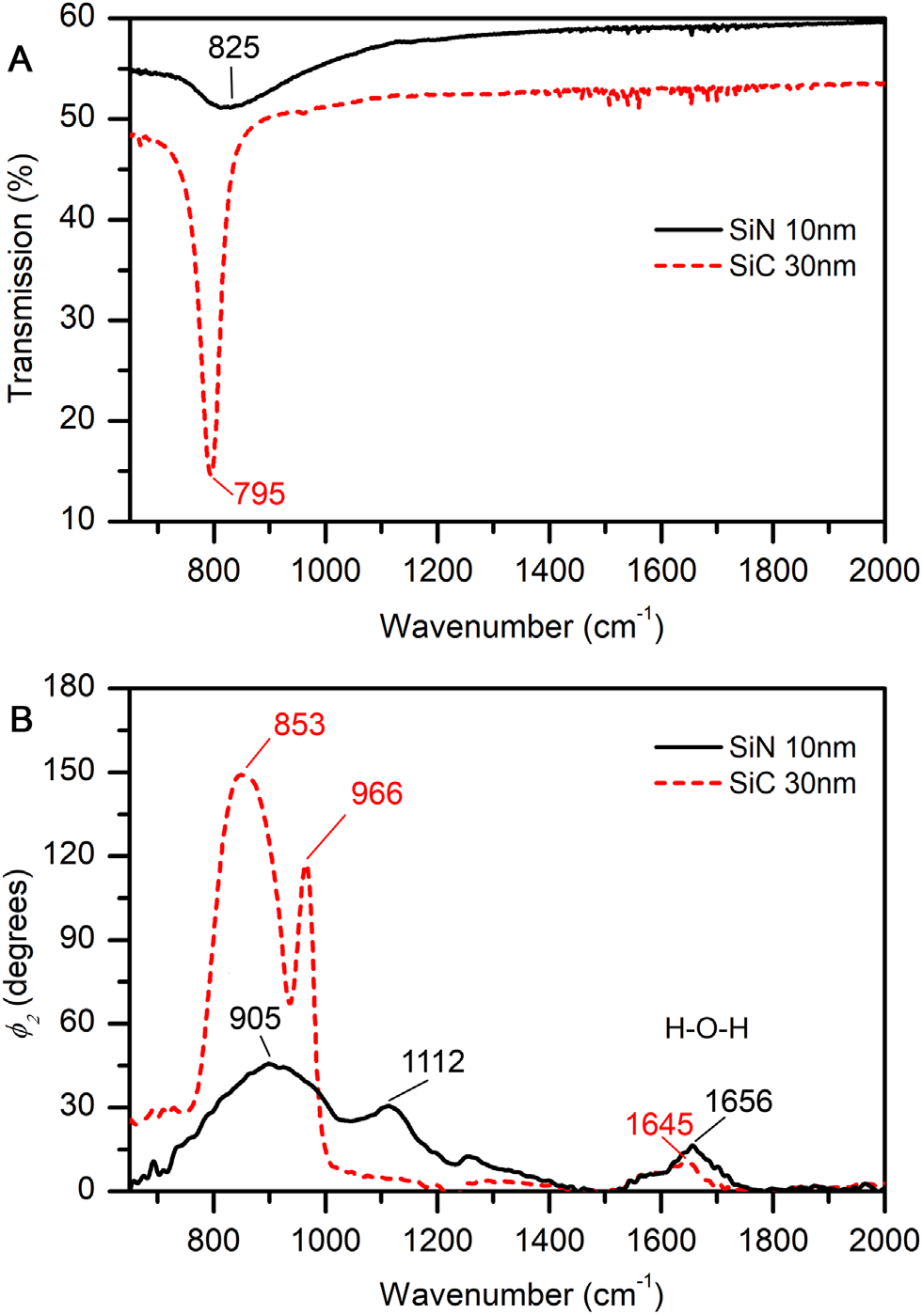
A) Far‐field transmission of 10 nm SiN and 30 nm SiC membranes. B) Nano‐IR spectra of PBS‐buffer measured using 10 nm SiN and 30 nm SiC membranes.

To characterize and compare the optical properties of the membranes used in the experiments, we measured both far field transmission and near‐field phase shift spectra of an SiN membrane and an SiC membrane of a thickness of 10 nm and 30 nm, respectively. The spectra are displayed in **Figure** **1**. According to the far‐field transmission spectrum, the SiC membrane is less transparent in the range from 1000  to 2000 cm^−1^ (Figure 1A), which is explained by the higher refractive index of SiC compared to SiN, with n_SiC_ ≈ 3 and n_SiN_ ≈ 2.3 at 2000 cm^−1^, leading to higher reflection losses. The intense phonon band of SiC is red‐shifted and is much sharper compared to the vibrational modes of SiN (Figure 1A). In the near‐field phase spectra of phosphate buffered saline (PBS) collected using the different membranes, a pair of well resolved vibrational bands is observed at ≈ 855 and 965 cm^−1^, and at 905 and 1110 cm^−1^ with the SiC and SiN membrane, respectively (Figure 1B). Both SiN and SiC are polar dielectrics, and surface phonon‐polaritons can be induced in the thin films made of these materials.^[^ ^27^ ^]^ The spectral response of the membranes in the range of ≈800–1000 cm^−1^ for SiC and 700–1400 cm^−1^ for SiN can be ascribed to the combination of the phonon and surface phonon‐polariton bands. Occurrence of the surface phonon‐polaritons in SiC and SiN thin films has been shown previously.^[^ ^28^, ^29^, ^30^, ^31^, ^32^, ^33^ ^]^ This explanation is also in agreement with previous results of simulations of the tip‐sample interactions using a multilayer dipole model that have allowed to explain the near‐field spectra of a free‐standing SiC membrane in air and to ascribe the two observed peaks to the phonon‐related TO‐mode and surface phonon polariton‐related resonance band.^[^ ^30^ ^]^

Even though the SiC membrane has a greater thickness than the SiN membrane, the lower energies of the phonon and polariton modes in SiC allow to achieve a broader transparency window in the IR s‐SNOM experiment, which in this case spans from ≈1000 cm^−1^ toward higher energies (Figure 1B, red trace). The band at ≈1650 cm^−1^ originates from the aqueous phosphate buffer underneath the membrane and is due to the bending mode of water.

### 2.2 IR Nanoimaging of Fibroblast Cells in Buffers and Cell Culture Media using an SiC Membrane

**Figure 2:**
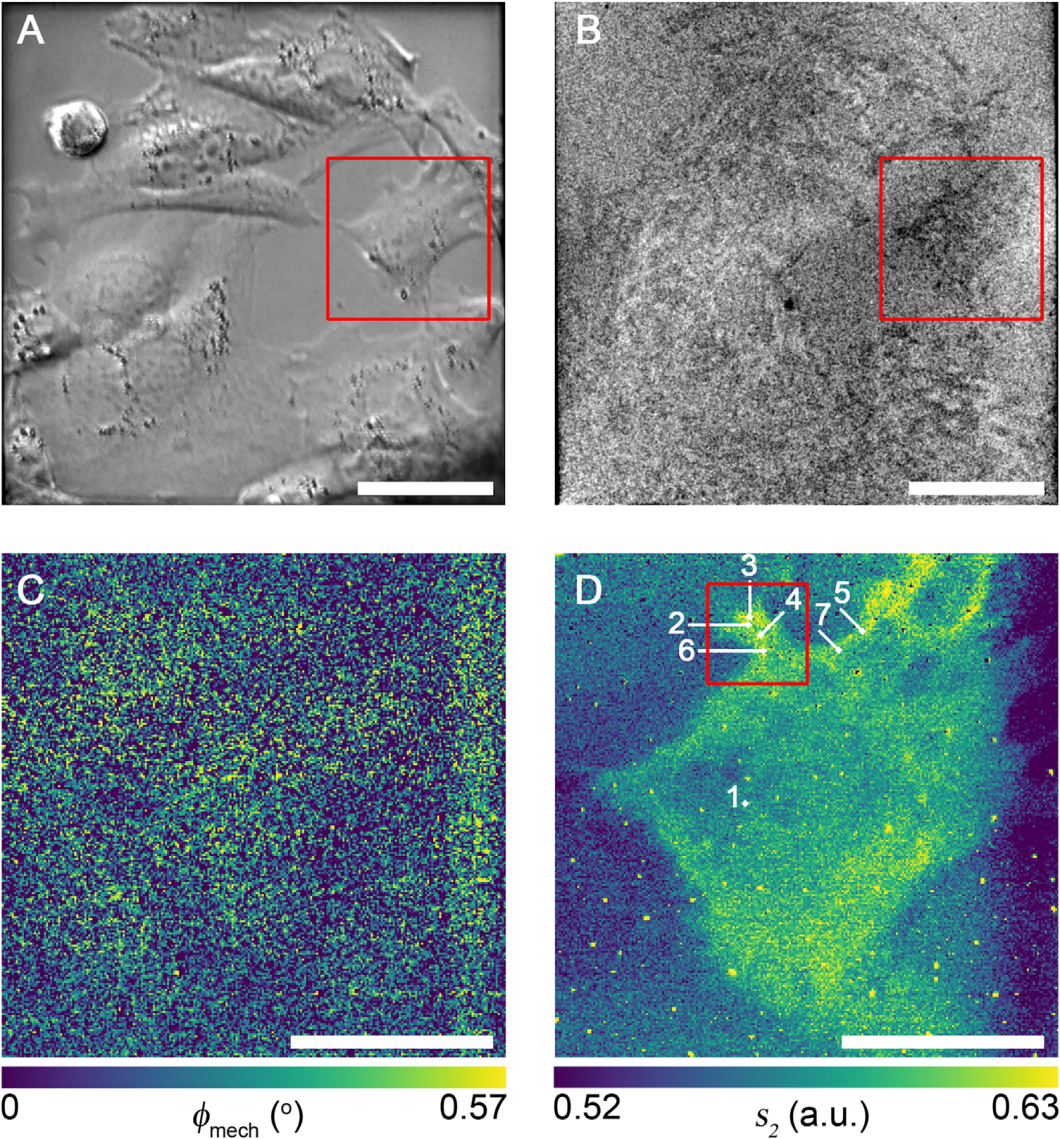
Images of the fibroblast cells in PBS buffer collected using 30 nm SiC membrane: optical microscope A) transmission and B) reflection images; C) AFM mechanical phase and D) near‐field optical amplitude images over the area indicated in panels (A,B). Scale bar in panels (A), (B) is 50 µm and in (C), (D) 20 µm. The labels in (D) indicate positions where individual nano‐IR spectra were collected. The corresponding spectra are shown in Figure 7A. The frame in panel (D) indicates a 10 × 10 µm^2^ area that was used for investigation of the influence of the tip‐sample interaction on IR nano‐imaging quality (cf. Figure 4).

An animal cell culture of the mouse fibroblast cell line 3T3 was grown on the membranes and found to adhere well. In the first feasibility studies, the samples were fixed with paraformaldehyde to circumvent potential detachment and immersed in phosphate buffered saline (PBS). The SiC is transparent in the visible range, which allows to use bright field microscopy for a preliminary characterization of the samples, in particular the first evaluation of cell morphology, to follow the growth process and enable orientation in the sample. After the transfer of the membrane to the sample holder, brightfield images of the cells were acquired in transmission mode (**Figure** **2**A). The cells grow to confluence on the membrane surface, indicating that the substrate can assure viability and adhesion. It is possible to distinguish different cells as well as sub‐cellular components, in particular the nuclei. In reflection mode, only cell shape can be distinguished, without providing more detail on cellular substructures (Figure 2B). Attaining higher contrast images in reflection proves difficult due to the high refractive index of SiC in the visible range of ≈ 3.3–3.5. Nevertheless, careful comparison of transmission and reflection images reveals that the areas and the borders of the cells do not match perfectly (compare Figure 2A,B). The contrast in reflection mode originates from thin‐film interference, and the cells are visible only when they are in contact with the membrane. An area with an entire fibroblast cell of this sample, as indicated in Figure 2A,B, was imaged using the s‐SNOM technique and the broadband synchrotron infrared (IR) “white” light. No changes could be detected in the mechanical phase (Figure 2C) or other mechanical AFM signals (not shown), in agreement with a rather high expected mechanical strength of the 30 nm thick SiC membrane.^[^ ^26^, ^34^ ^]^ At the same time, the fibroblast cells are well visible in the near‐field IR optical image as shown by the example in Figure 2D. The shape of the cell matches well the optical micrograph and allows to resolve the cellular morphology with much better spatial resolution. We found that the s‐SNOM images depend on the contact between the cell and the SiC membrane. We ascribe the darker areas in Figure 2D to spots with weaker adhesion of the cell to the membrane. An inhomogeneous adhesion of a cell can make a characterization of its morphology based on near‐field images difficult. In the example shown in Figure S1 (Supporting Information), the alternating regions of strong and weaker adhesion within a single fibroblast cell are in agreement with the known heterogeneity of the cellular membrane structure and the extracellular matrix.^[^ ^35^ ^]^

**Figure 3:**
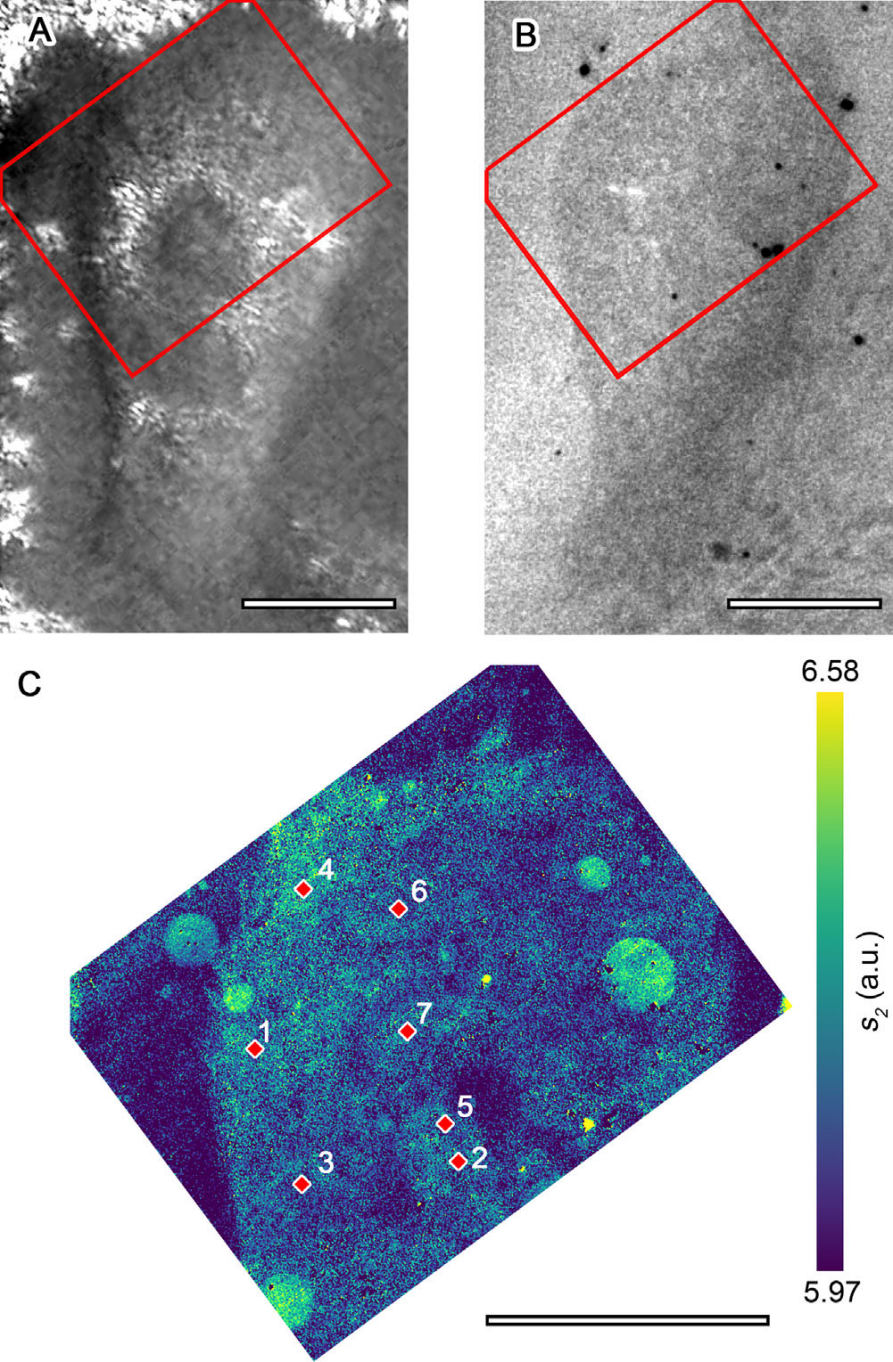
Images of a fibroblast cell in culture medium collected using a 30 nm SiC membrane: optical microscope A) transmission and B) reflection images. C) near‐field optical amplitude image over the area indicated in panels (A,B). Scale bars: 30 µm. The labels in panel (C) indicate positions where individual nano‐IR spectra were collected. The corresponding spectra are shown in Figure 7B.

The in‐liquid IR nano imaging and spectroscopy experiment was also carried out with live fibroblast cells in their complete culture medium. The transmission, reflection, and the near‐field IR images of the cell are shown in **Figure** **3**A–C, respectively. Also here, the optical transmission and reflection micrographs indicate good cell adhesion, and more importantly, the white light nano‐IR image demonstrates significant contrast between the environment and the cell.

### 2.3 Influence of the Tip‐Sample Interaction on IR Nano‐Imaging Quality

In our experiment, we found that the SiC membrane allows to significantly vary the oscillation parameters of the AFM probe without compromising the stability of the tip‐sample interaction. The mechanical interaction on the SiC membrane was stable for the in‐contact oscillation amplitude of the cantilever (hereinafter referred to as “tapping amplitude”) in the range of 20 –130 nm and setpoint values from 55 % to 85 %. This is in contrast to previous reports on SiN membranes, where the use of low tapping amplitudes, for example of 40 nm, often resulted in trapping of the cantilever on the sample.^[^ ^36^ ^]^

The optimal tapping amplitude is often chosen so that the highest spatial resolution and the best contrast between different materials or phases in the X‐Y plane are reached.^[^ ^37^ ^]^ With this as the goal, a tapping amplitude comparable to the AFM probe tip radius often ensures close‐to‐optimal conditions. In this case, the decay depth of the optical near‐field signal is comparable to the AFM tip radius for the second harmonic of the signal demodulation.^[^ ^37^ ^]^ Here, a tip with a radius of 25 nm enabled us to study our samples through the 30 nm SiC membrane. This is in agreement with previous work, indicating that the signal could come from a larger depth of up to 100 nm in this case.^[^ ^38^ ^]^ The use of higher tapping amplitude and lower set‐point values could increase the probing depth of the technique, due to increased signal decay depth.^[^ ^38^ ^]^ However, too high tapping amplitudes could also increase the background signal contribution.^[^ ^37^, ^38^, ^39^ ^]^ The probing depth and the background signal contribution are two competing factors that both influence the quality of the data acquired in the near‐field experiment.

**Figure 4:**
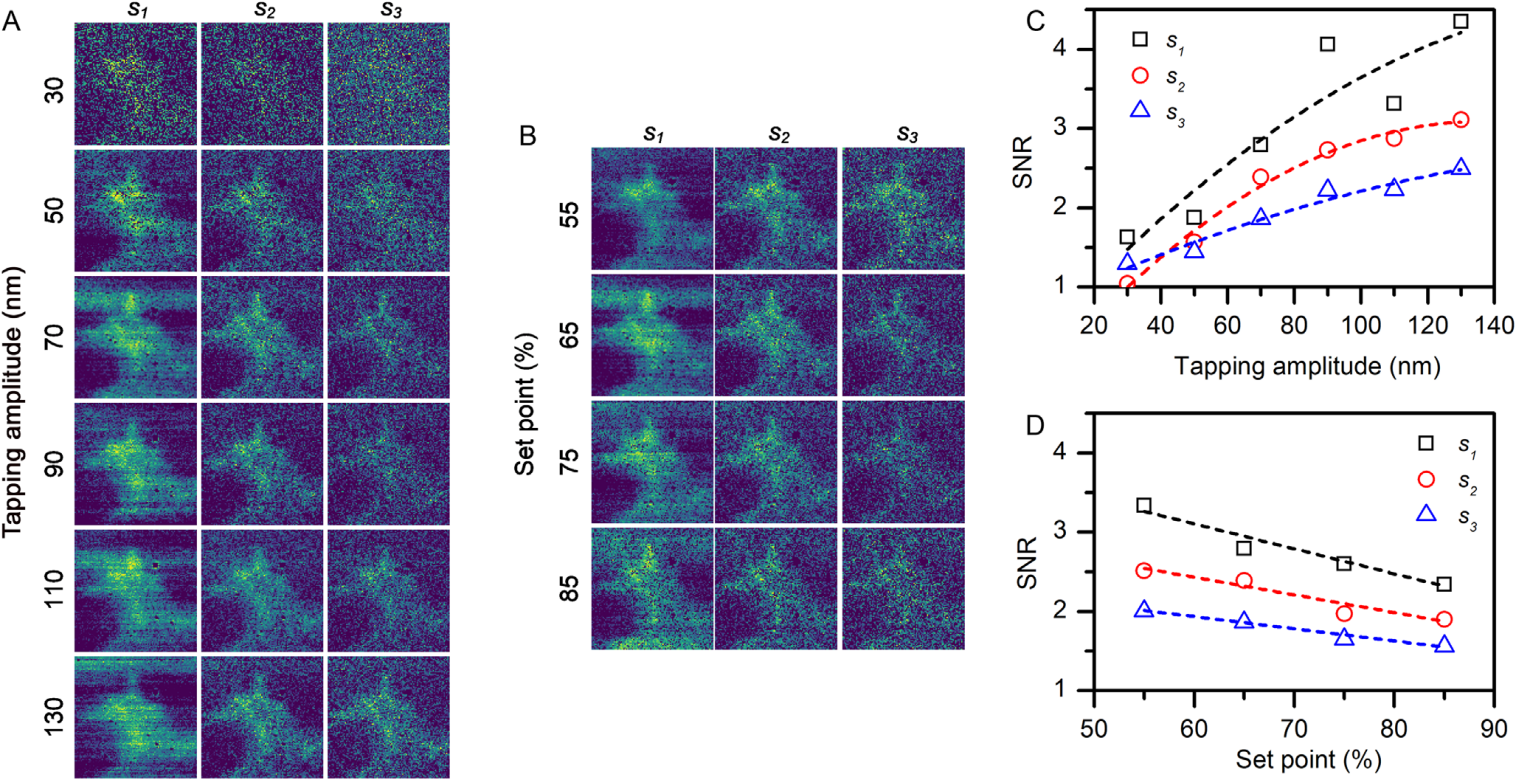
Near‐field IR optical amplitude images of a fibroblast cell area of 10 × 10 µm^2^ (indicated in Figure 2D) underneath the SiC membrane collected using different AFM‐probe oscillation parameters: A) using different tapping amplitudes at the constant set‐point value of 65%; B) using different set‐point values at the constant tapping amplitude of 70 nm. Images acquired for 1st, 2nd, and 3rd demodulation harmonics of the optical amplitude signal are shown in *s_1_*, *s_2_*, and *s_3_* columns, respectively. C,D) The corresponding signal to noise ratio (SNR) was calculated using the images in panels A and B, respectively: (C) SNR versus the tapping amplitude at the constant set‐point value of 65%; (D) SNR versus the set‐point value at the constant oscillation amplitude of 70 nm. The lines are guides to the eye only.

We studied the influence of the AFM probe oscillation settings on the quality of the white‐light nano‐IR images of a cell collected through the SiC membrane. Almost all images display the morphology of the fibroblast, with the large cell body, but the visibility of the protruding filopodia that the cells use to sense and interact with their growth substrate, in this case the SiC membrane, differ depending on the settings. **Figure** **4** shows that both the tapping amplitude (Figure 4A) and the set‐point (Figure 4B) significantly influence the near‐field images generated using the 1st, 2nd, and 3rd demodulation harmonics of the optical signal (see columns *s_1_*, *s_2_*, *s_3_* in Figure 4, respectively). Their quality increases when a higher tapping amplitude and a lower set‐point are used. The yielded significant increase in the signal‐to‐noise ratio (SNR), allows imaging in the 3rd demodulation harmonics of the signal (column *s_3_* in Figure 4A,B). Figure 4C,D shows the SNR calculated for the s‐SNOM images of Figure 4A,B, respectively. The SNR was calculated using the integral signal over the areas with and without the cell, as explained in Figure S2 (Supporting Information). With increase of the tapping amplitude from 30  to 130 nm, the SNR for the *s_2_* signal (Figure 4C, red circles) increases from a value of 1 (the signal is equal to the noise) to a value of 3 at the constant set point of 65%. The decrease of the set point from 85% to 55% at the constant tapping amplitude of 70 nm increases the SNR of the *s_2_* signal from 2 to 2.5 (Figure 4D, red circles). Similar trends are observed for the 1st and the 3rd demodulation harmonics of the optical signal as well (Figure 4C,D, blue triangles and black squares).

It worth noting that the cells are well visible in the 1st demodulation harmonic of the optical amplitude signal acquired by the s‐SNOM setup (see *s_1_* column in Figure 4A,B). It is known that the higher demodulation harmonics are required to suppress significantly the background contribution and emphasize the near‐field part in the overall optical signal.^[^ ^39^ ^]^ The high quality of the 1st harmonic images therefore indicates that the background optical signal level is quite constant. This indicates a good stability of the synchrotron IR‐beam, of the temperature, and the mechanical stability of the set‐up, and the close‐to‐ideal roughness of the sample, in this case the SiC membrane. In this way mainly the near‐field signal varies and contributes to the s‐SNOM image.

Our results show that the SNR still increases for tapping amplitudes at least up to 130 nm, so that the increase of the actual near‐field signal is prevalent over the additional background signal contribution. The improvement of the signal due to the lowering of the set‐point could be explained by a closer approach of the AFM‐probe to the surface of the membrane, which allows to collect the signal from the deeper volume of the sample.

**Figure 5:**
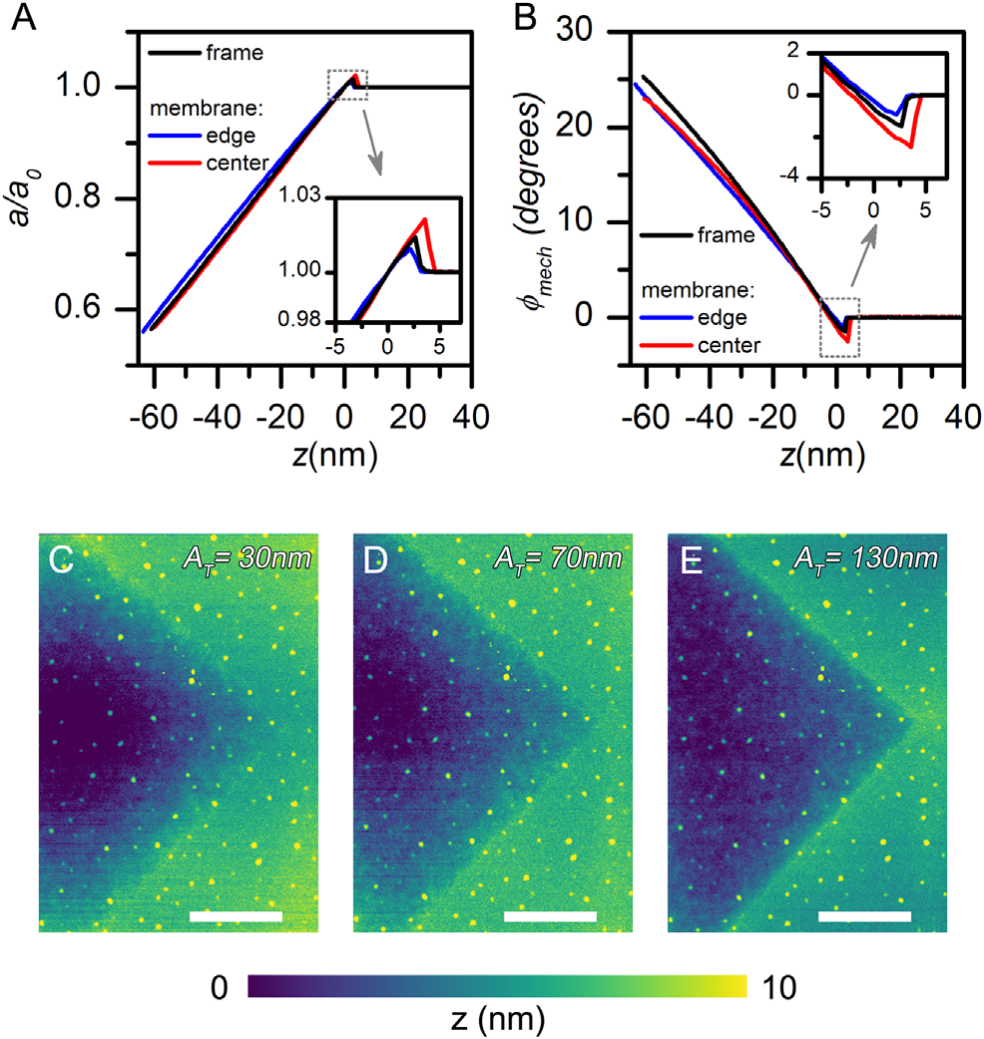
Tapping‐mode retract curves from the 30‐nm SiC membrane on the membrane holder frame, and membrane close to the edge of the frame and close to the center: A) normalized tapping amplitude, *a/a_0_*; B) tapping phase 𝜑*_mech_*. Insets in panels (A,B) show the corresponding traces near the tip–sample contact point. C–E) Topography of the membrane near the membrane‐frame corner measured at tapping amplitudes (*A_T_*) of (C) 30 nm, (D) 70 nm, and (E) 130 nm. Scale bar in panels C–E is 10 µm.

Another aspect in the discussion of the near‐field signal enhancement with increase of the tapping amplitude and decrease of the set point is the local deformation of the ultra‐thin membrane, allowing to non‐destructively indent and locally “immerse” the tip into the sample. Recently, adjustment of the mechanical interaction between the AFM‐probe and a 10 nm SiN membrane allowed to manipulate the ultrathin membrane and enhance the infrared near‐field microscopy of liquid‐immersed samples through a reversible nanometric deformation of the membrane.^[^ ^36^ ^]^ As the SiC membrane used in the current experiment is thicker, and SiC has a higher Young's modulus and fracture toughness than SiN, significantly less deformation of the membrane should occur. Nevertheless, we cannot exclude the effect of higher local pressure from the used AFM‐probe tips, as they have a smaller radius than those used in the discussed work.^[^ ^36^ ^]^ To understand the significance of membrane deformation in the experiment with 30 nm SiC membranes and 25 nm tip‐radius AFM probe, we collected retract curves from the center of the membrane, from its edge at the distance of ≈2 µm from the membrane frame, and at the rigid SiC/Si frame and found that the data from the center differ significantly (see **Figure** **5**A,B). This agrees with the previous work reported for an SiN membrane and could originate from the adhesion of the AFM probe on the membrane.^[^ ^36^ ^]^

Nevertheless, a topography image collected close to the edge between the rigid frame and the 30 nm SiC membrane does not show significant variation upon change of the tapping amplitude (Figure 5C–E). The maximum height variation between the frame and the deepest region of the membrane in the image does not show significant dependence on the tapping amplitude. The height differences measured from the collected images are 8, 8, and 6 nm for the cantilever oscillation amplitudes of 30, 70, and 130 nm, respectively. Moreover, the retract curves measured close to the edge of the membrane in the *z*‐region close to the contact point only slightly differ from the trace collected at the frame (see insets of Figure 5A,B, blue and black curves).

To better understand this behavior, we collected retract curves from the frame, the central, and edge regions of the membrane, using AFM contact mode (see Figure S3, Supporting Information). These traces reveal significant membrane deformation, with greater deformation occurring at the center and less near the edge — as intuitively expected when a point force is applied to either the center or the edge of a thin membrane. The inconsistency between amplitude–distance curves obtained in contact and tapping AFM modes highlights the complexity of tip–membrane mechanical interactions under intermittent contact conditions. The difference in the retract curves collected at the center and at the edge of the membrane suggests that the force applied by the AFM probe can result in deformation of the membrane, however to the large extent this can be a deformation of the membrane as a whole and not a significant local indentation/bulging of the membrane at the nanometer scale.

The control of the tip‐sample mechanical interaction can be used to establish a method for s‐SNOM based in‐liquid nano‐tomography. As indicated by our data, the control of the tapping amplitude and the set point can be used to gradually vary the probing depth in the s‐SNOM method. This intuitive understanding can also be demonstrated by converting the SNR plots versus amplitude and the set point (Figure 4C,D) into a single plot of SNR versus tip–sample distance (see the corresponding section and Figure S4A–D, Supporting Information). From Figure S4D (Supporting Information), one can see that both oscillation parameters enable direct control over the tip‐sample distance and systematic variation of the near‐field signal probing depth. This effect can be used to reconstruct the chemical composition of the sample in depth using the optical signal from a *single* demodulation harmonic. This approach differs from another suggestion to achieve nano‐tomography with nano‐IR via the analysis of the different demodulation harmonics of the signal that was reported earlier.^[^ ^40^ ^]^ The examination of the influence of the tip‐sample interaction on the nano‐IR imaging done in this work indicates that the use of a higher tapping amplitude and lower set‐point values enables the collection of the signal from greater depths. In this approach, the collection of the signal from greater depths of the sample deteriorates the in‐plane spatial resolution of the method, due to the broadening of the electrical field with distance from the AFM‐tip. Nevertheless, even nano‐tomograms of 100 nm spatial resolution (in X‐Y plane) collected in liquid medium can provide valuable information about biological objects, like single cells, fibers, and extracellular matrix in tissues etc. in native environments.

### 2.4 Nano‐IR Spectra of the Cells in Aqueous Solutions

The SiC membrane enables the collection of nano‐IR spectra in the range down to 1050 cm^−1^, which is determined by the absorption bands of the membrane material (cf. Figure 1).

**Figure 6:**
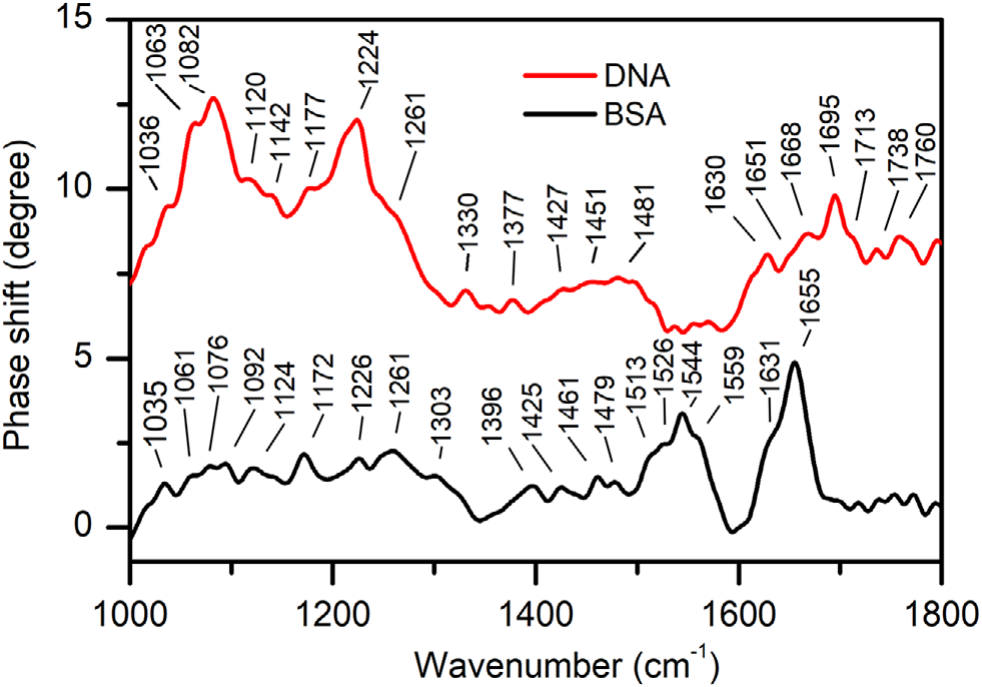
Nano‐IR spectra of DNA and BSA aqueous solutions.

**Figure 7:**
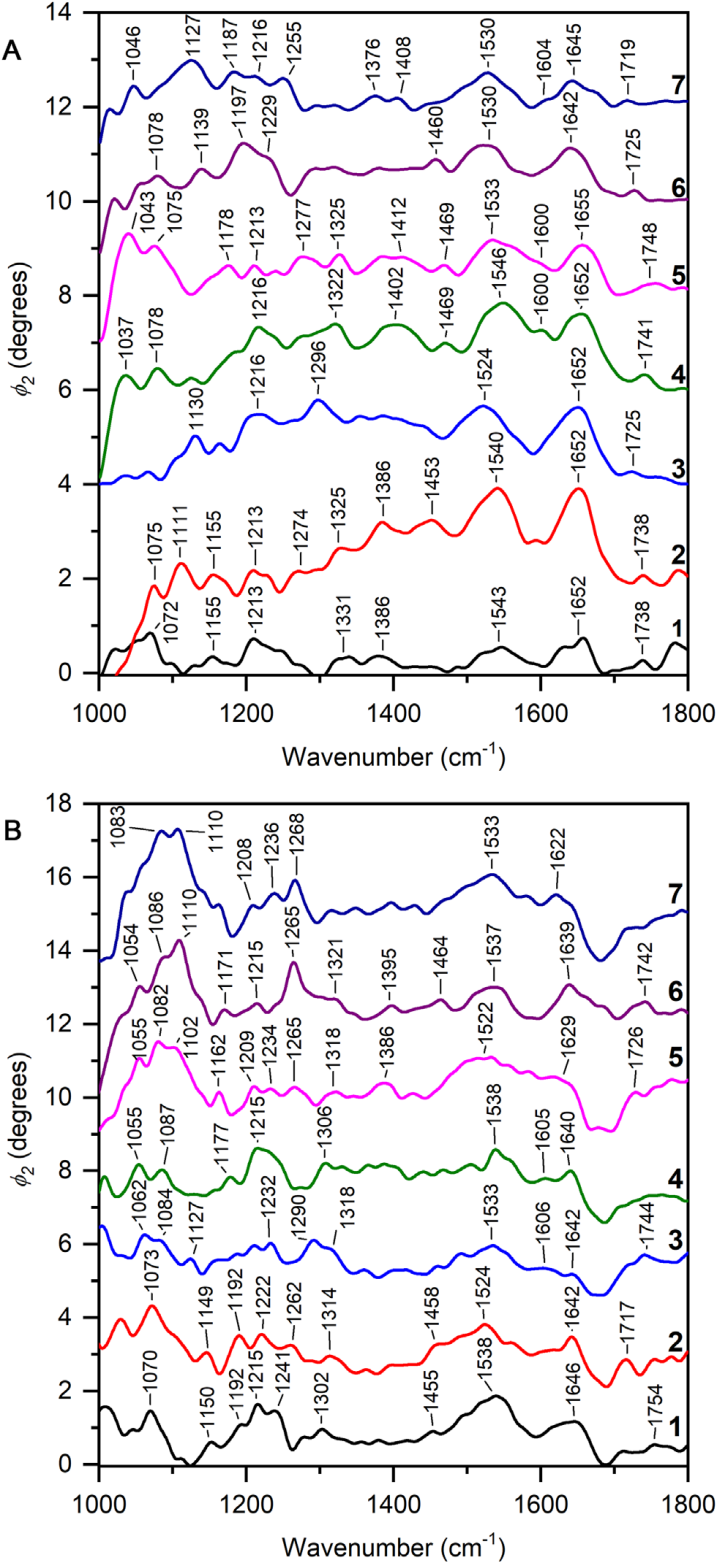
Nano‐IR spectra collected at different points of interest from A) a fixed fibroblast cell in phosphate buffer and B) a live fibroblast cell in culture medium. The numbering of the spectral traces in panels (A,B) corresponds to the points in Figures 2D and 3C, respectively.

To simplify the situation of the complex mixture of biomolecules in solution in an animal cell, we collected spectra of DNA and the protein bovine serum albumin (BSA) molecules in aqueous solutions (**Figure** **6**). The assignments of the vibrational bands are displayed in Table S1 (Supporting Information) and discussed there. Spectra were also collected at different locations within cells immersed in PBS buffer, and from live cells in culture medium. The exact positions selected for the nano‐IR spectroscopy experiment following the imaging of a whole cell are marked in Figure 2D, and in Figure 3C for examples of fixed and live cells, respectively. **Figure** **7** shows the corresponding spectra, and the tentative assignments of the observed vibrational bands are given in **Table** **1**.

**Table 1:** Tentative assignment of the vibrational bands observed in the spectra of the fibroblast cells using micro‐IR and nano‐IR spectroscopy in dry and liquid environment, respectively. The assignment is based on the data reported in the literature.^[^ ^44^, ^47^, ^50^, ^51^, ^52^, ^53^, ^54^, ^55^, ^56^ ^]^

Wavenumber [cm^−1^]		Tentative band assignment
micro‐IR	nano‐IR^a)^	
1061		C─O, C─O─C stretching, carbohydrates
1086	1075 (1070–1080)	PO_2_^−^ symmetrical stretching, DNA
	1107 (1102–1111)	C─O stretching, carbohydrates
1124	1130 (1127–1139)	C─O stretching, carbohydrates
1236	1213 (1208–1222)	PO_2_^−^ asymmetrical stretching, DNA
	1235 (1232–1240)	Amide III / PO_2_^−^ asymmetrical stretching, proteins/DNA
	1293 (1274–1296)	Amide III, proteins
	1325 (1318–1325)	Amide III, proteins
	1386 (1376–1386)	CH_3_ bending, lipids
1394	1408 (1395–1412)	COO^−^ symmetric stretch, fatty acids, and amino acids
1456	1456 (1453–1469)	CH_2_ deformation, lipids, and carbohydrates
1541	1533 (1522–1546)	Amide II, proteins
1655	1652 (1622–1652)	Amide I, proteins
	1738 (1726–1748)	C═O ester stretching, lipids

^a)^ the values in parentheses indicate the possible variation of the band position between individual nano‐IR spectra.

In all spectra collected from the cells (Figure 7), the bands related to amide I (1622–1652 cm^−1^) and amide II (1522–1546 cm^−1^) vibrations are among the most prominent, and together with signals assigned to amide III (multiple bands in the range 1232–1325 cm^−1^) related modes suggest the presence of proteins, in agreement with the spectrum of the pure protein in solution (Figure 6). Also signals related to carbohydrates, such as the C─O stretching modes at 1055  and 1110 cm^−1^, and of phosphate stretching vibrations at ≈1080  and ≈1215 cm^−1^ of DNA, as well as of lipids (e.g., CH_2_ deformation at 1455 cm^−1^ and C═O stretching between 1730 and 1750 cm^−1^) are found. It should be noted that due to the strong vibrational modes of the SiC the baseline correction is challenging in the range of 1000–1050 cm^−1^.

Overall, significant variation in the relative peak intensities related to variations in concentration of proteins, DNA, RNA, lipids and carbohydrates is observed between the spots, indicating on the local variation in chemical composition within the cells in nanometer‐scaled volumes. As examples, the bands related to lipids, such as the C═O ester stretching mode at 1738 cm^−1^, the CH_2_ deformation vibration at 1453 cm^−1^, and the CH_3_ bending mode at 1386 cm^−1^ can be clearly observed in spectrum 2 of Figure 7A, but are absent or have lower relative intensity in spectrum 3 (compare the corresponding traces in Figure 7A). The corresponding spots are separated by approximately one micron (cf. Figure 2D). Such differences in lipid composition have been recently found for this cell type in vibrational spectra that we have obtained by surface‐enhanced Raman scattering (SERS) from the membrane of cells of this cell line^[^ ^35^ ^]^ that underpin its micrometer‐sized heterogeneity.^[^ ^41^, ^42^ ^]^ Bands related to carbohydrate C─O stretching modes have different relative intensities in the spectra 2, 4, and 6 of Figure 7A, with the corresponding spots within a 2.5 µm radius (cf. Figure 2D), suggesting the presence of different polysaccharides or their different interactions with other molecules. Spectrum 1 is collected from a spot located in the vicinity of the nucleus (cf. Figure 2D) and demonstrates significantly lower relative intensity of the protein bands with respect to other vibrational modes (cf. Figure 7A).

The differences found between the spectra of paraformaldehyde‐fixed cells in aqueous buffer (Figure 7A) and the spectra from the live cells in culture medium (Figure 7B) are consistent with the effects of this fixative discussed based on conventional FTIR microspectra.^[^ ^43^, ^44^ ^]^ They include, e.g., changes in the intensity ratio of the amide I (∼1640 cm^−1^) and amide II (∼1530 cm^−1^) bands with other signals. Paraformaldehyde is known to crosslink proteins and creates surface clusters of membrane proteins in mammalian cells.^[^ ^45^ ^]^

The chemical composition and structure, and the localization and heterogeneity of sample biochemistry that is revealed in the nano‐IR spectra reported here for the case of the cell membrane and cellular substructures near the membrane agrees well with that obtained from SERS experiments from the same cell line.^[^ ^35^ ^]^ Nevertheless, the complementarity of both vibrational near‐field approaches becomes obvious when we compare the relative contributions of modes from different molecular classes. Specifically, the signals of proteins in the amide I and amide II regions are pronounced in the nano‐IR spectra but are usually weaker in SERS experiments.

Overall, the IR spectra collected via the in‐liquid s‐SNOM technique are similar to the spectra that are usually acquired by far‐field IR spectroscopy techniques. The individual nano‐IR spectra displayed in Figure 7A were used to calculate an average spectrum. The calculated nano‐IR spectrum shares several similarities with an FTIR spectrum collected from a dry 3T3 fibroblast cell collected using transmission IR microscopy (Figure S5, Supporting Information). This concerns several of the bands assigned to the amide I and amide II vibrations and signals from DNA. Nevertheless, the average and the corresponding individual nano‐IR spectra reveal much more clearly visible vibrational modes, including phosphate and ester carbonyl signals from lipids that otherwise “average out”.

The differences in the peak positions and relative intensities between the far‐field and the near‐field IR spectra can be explained by further factors. The s‐SNOM measurement in this work was done in native liquid state of the cell, and it is known that the hydration level strongly influences the FTIR spectra observed from different components of the cell.^[^ ^46^, ^47^ ^]^ For example, the antisymmetric stretching of DNA‐related PO_2_^−^ phosphate groups shifts from ≈ 1220 to 1240 cm^−1^ upon dehydration, simultaneously with significant enhancement of the symmetric stretching mode of PO_2_^−^ group at ≈ 1080 cm^−1^.^[^ ^47^ ^]^ These two characteristic DNA bands are also observed in the spectra of pure DNA in water measured with the SiC membrane (Figure 6). Thus, the difference in the hydration level between the near‐field and the far‐field experiments could explain the higher intensity of the DNA modes and the difference in the positions between the far‐field absorption and the nano‐IR spectra. More importantly, the near‐field spectra do not always match the far‐field data due to the different mechanisms of the sample and IR‐light interaction. The near‐field interaction of the dipole induced in the AFM tip with the sample often leads to different relative intensities of the spectral bands and significant spectral shifts.^[^ ^48^ ^]^

### 2.5 Use of SiN Membranes Instead of SiC Membranes for Nano‐IR Characterization of Cells

To compare the applicability of SiN and SiC membranes for the in‐liquid IR nano spectroscopy and imaging of fibroblasts we also carried out the experiment using 10 nm SiN membranes of the same type as were used in a previous study.^[^ ^24^ ^]^ We found that both materials are biocompatible after an air‐plasma and UV treatment, and that fibroblast cells adhere well and can be grown directly on SiN surface (Figure S6A,B, Supporting Information). In our experiments we could not detect significant differences in the cell growth and adhesion to the membrane between the SiC and SiN substrates, different from a previous study of biocompatibility of these materials.^[^ ^26^ ^]^ In transmission mode visible bright field microscopy (Figure S6A, Supporting Information) it is possible to distinguish conveniently different cells as well as cellular compartments, in particular the nuclei. Due to the lower refractive index of SiN in the visible range, images captured in the reflection mode using SiN membranes are of higher contrast than SiC membranes, which simplifies the recognition the cellular morphology (Figure S6B, Supporting Information). The nano‐IR images allow to resolve the morphology of the cell‐membrane interface with high spatial resolution and match well the optical microscopy data (cf., e.g., regions R1 and R2 in Figure S6A, Supporting Information, with Figure S6E,G, Supporting Information, respectively). Similar to use of the SiC membranes, the observed s‐SNOM images also depend on the contact between the cell and the SiN membrane. Due to the small thickness, the fibroblast cells have some influence on the topography (Figure S6C, Supporting Information) and mechanical (Figure S6D, Supporting Information) AFM signals registered over the area. We did not note differences in the near‐field images’ quality collected using the 10 nm SiN and the three times thicker (30 nm) SiC membranes (compare, e.g., Figures 2D and 3C; Figure S6C,D, Supporting Information). Although previously, it was shown that in layered systems, the signal decreases with increase of the top layer thickness, the decrease rate within a material is much lower than it would be in the presence of an air gap between the tip and the sample.^[^ ^38^, ^49^ ^]^ This supports the possibility of the near‐field optical imaging through the thicker SiC membrane that is more rugged with respect to the cell culture application and handling.

The nano‐IR spectra collected at different spots of the fibroblast cells using the SiN membranes are shown in Figure S6E (Supporting Information). The spectral features observed from the fibroblast cells using the two different substrates agree well (compare Figure S6G, Supporting Information with Figure 7A). Also, in the cells grown and probed on SiN membranes, the dominant bands can be assigned to proteins. Due to the presence of vibrational bands in the range of 800–1200 cm^−1^ originating from the SiN membranes (cf. Figure 1B), this spectral region should be used for spectroscopy of the cells with special care. We found that the referencing against the background signal in this spectral range often leads to non‐linear baseline, significant variation in the relative intensities of the spectral bands, and hence potential spectral artefacts. The signals in the spectra vary significantly between the different sampled spots across the cells, some demonstrate much weaker spectral features than others (compare, e.g., spectrum 1 and 2 with the other spectra in Figure S6G, Supporting Information), which we ascribe to weaker local adhesion of the cell to the SiN membrane in these positions.

## 3 Conclusion

As demonstrated by the data, we advanced in‐liquid IR s‐SNOM measurements toward an acquisition of nano‐IR images and spectra from biological samples through an ultrathin membrane and applied this to study living fibroblast cells in cell culture media. It was shown that both SiN and SiC membranes can be used as substrates for the growth of adherent animal cells. A direct growth procedure was established that enabled good attachment of the cells to the substrate. This factor was found to be critical for s‐SNOM based nano‐IR imaging and spectroscopy. The generation of optical amplitude images using broadband IR‐synchrotron radiation and their comparison with mechanical phase images demonstrated that the nano‐imaging of cells and sub‐cellular structures in‐liquid via s‐SNOM is possible. The results show that the method is sensitive to the differences in the chemical composition between the cell and the outside medium, ensuring biocompatible conditions for the cells during the experiment. We found that optimization of the tip‐sample mechanical interaction allows to significantly increase the signal‐to‐noise ratio of the collected data. Specifically, the control of the AFM probe oscillation amplitude and set‐point paves the way to a new approach of s‐SNOM nano‐tomography using a single demodulation harmonic signal. Since the near‐field optical signal in the s‐SNOM experiment is directly connected to the mechanical interaction between the AFM‐probe and the ultra‐thin membrane the deeper understanding of all its aspects would enable more reliable and better interpretable near‐field in‐liquid experiments.

Similarity of the IR spectra collected by in‐liquid s‐SNOM to the far‐field IR spectra allows to interpret the nano‐IR data using the knowledge about the IR response of the biomolecules and cells. The nano‐IR spectra collected at different spots of the same cell demonstrate significant variation, indicating a local variation of the chemical composition at the nanoscale. This is in agreement with earlier work using other nanoscale vibrational approaches to characterize the biochemical heterogeneity of the cells, for example in the composition of their membrane. The comparison of the spectra with those of pure DNA and protein illustrates that standardization of the in‐liquid s‐SNOM technique and collection of nano‐IR spectra of the basic compounds will help to interpret cell spectra in future applications.

Our brief comparison showed that both SiC and SiN ultra‐thin membranes are suitable for in‐liquid nano‐IR imaging and spectroscopy experiments. However, the use of SiC is preferential to enable experiments in a broader spectral range and ensures a higher reliability of the spectral data in the range below 1200 cm^−1^. The spectral benefit applies for both synchrotron‐ and IR laser‐based experiments. However, the unique spectral broadness of synchrotron IR radiation enables full coverage of the available spectral range in a single measurement. In the future, using a thinner, e.g. 10 nm, SiC membrane may further extend the accessible spectral range toward lower frequencies. Additionally, the use of appropriate detectors in combination with the synchrotron radiation will facilitate the experiments in the far‐IR spectral region.

The possibility to obtain both high quality nano‐IR spectral information as well as high contrast in broadband IR imaging from living cells in media and buffers suggests a further refinement of the acquisition regimes. It will include the selection of optimal vibrational frequencies for fast single‐ or few‐frequency imaging, for example using a tunable quantum cascade laser. The significant differences observed in nano‐IR spectra confirm that imaging at characteristic frequencies will enable measurement of the spatial distribution of a broad range of biomolecules within a single cell or other complex bio‐materials. This will be a further step to fast hyperspectral nano‐IR imaging of animal cells and other materials of biological and organic materials, such as hydrogels and polymers, and reactions at liquid interfaces.

## 4 Experimental Section

### Membrane Preparations and Cell Growth

Two types of ultra‐thin membranes were used for the experiments: 250 µm x 250 µm x 10 nm SiN (NX5025Z, Norcada Inc., Canada), 200 µm x 200 µm x 30 nm SiC (Silson Ltd., UK). Both the membranes are supported by a 5 × 5 mm Si frame, which allows to use them in the same sample holder designed in house.

Solutions of 0.55 g mL^−1^ bovine serum albumin (BSA; Sigma–Aldrich, Steinheim, Germany) and 1% DNA (low molecular weight from salmon sperm; Sigma‐Aldrich, St. Louis, USA) were prepared in MilliQ water.

To improve hydrophilicity of the membranes before the cell growth the membranes were treated via air plasma cleaning for 10 min and exposed to UV‐C light for another 20 min.

Swiss albino mouse fibroblast cell line 3T3 (DSMZ, Braunschweig, Germany) was grown in DMEM with Phenol Red (Bio&SELL, Nuremberg, Germany) supplemented with 10% Fetal Calf Serum (Fisher Scientific, Schwerte, Germany) and maintained in a humidified environment at 37 °C with 5% CO_2_. For the s‐SNOM experiments, cells were grown on plasma and UV‐C treated SiN or SiC membranes for 24 h. Cells were then washed with PBS (Sigma‐Aldrich, Steinheim, Germany) twice and fixated using paraformaldehyde 4% in PBS (Affymetrix, Cleveland, USA) for 20 min at 37 °C. After fixation, cells were washed again with PBS twice and stored in the same buffer at 4 °C until measurement. For live cell measurements, cells were grown in a similar fashion and kept in the serum‐containing culture medium during measurements.

To prevent contact between the outer side of the SiC or SiN membrane and the culture medium during cell growth, a silicone protective cap was used (Press‐to‐Seal Silicone, Invitrogen‐Thermo Fisher Scientific, Schwerte, Germany). The cap was made of two ca. 5 × 5 mm^2^ pieces, where the piece attached to the membrane frame had a hole of ≈ 3 mm to avoid direct contact of the silicone with the SiN or SiC membrane. The cap provided good attachment to the membrane frame, strong sealing against buffer leakage, and easy removal when transferring the membrane to the sample holder.

The sample holder was made of a small Al‐block with a 3 mm hole in the middle. A thin layer (≤50 µm) of a neutral transparent sealant was used to fix the membrane via its frame on the sample holder. The back side of the holder was sealed with 50 µm polyethylene film, which was glued using the thin layer of the sealant.

### Sample Preparation for Comparison Transmission IR Microscopy Experiment

Cells were seeded onto UV‐C treated CaF_2_ windows of 1 mm thickness and grown to confluence under standard conditions. They were then washed in PBS buffer. Subsequent rinsing with ultrapure water was done in upright position of the window and rapid transfer into an evacuating desiccator where the samples were dried immediately. The samples were stored in a desiccator.

### Infrared Nano Spectroscopy and Imaging Experiment

IR nano spectroscopy and imaging experiments were done at the IR‐nanospectroscopy end‐station, IRIS beam‐line, BESSY II synchrotron,^[^ ^57^ ^]^ using a scattering‐type scanning near field optical microscope (s‐SNOM) setup (neaScope, Attocube, Haar, Germany) coupled to the brilliant broadband synchrotron infrared light.^[^ ^58^ ^]^ The measurements were performed in amplitude‐modulation AFM mode, widely known as tapping mode, using two types of Pt‐Ir coated AFM‐probes: 1) AFM‐probes with typical tip radius <25 nm, nominal spring constant of 42 N m^−1^, and a resonance oscillation frequency of ≈ 255 kHz (Arrow NCPt, NanoWorld, Neuchâtel, Switzerland); 2) AFM‐probes with typical tip radius <50 nm, nominal spring constant of 37 N m^−1^, and a resonance oscillation frequency of ≈ 300 kHz (ANSCM‐PA5, Applied NanoStructures Inc., Mountain View, CA 94 043 USA). The cantilever was driven close to its resonance oscillation frequency. Most of the results in this work were obtained using Arrow NCPt cantilevers, the thicker ANSCM‐PA5 AFM‐probes were used for the live cell experiment (the data shown in Figures 3C and 7B) and nano‐IR spectroscopy of DNA and BSA water solutions (Figure 6).

Nano‐IR imaging was performed in zero‐path difference heterodyne imaging (known as “white light” imaging) mode using the broadband infrared synchrotron radiation.^[^ ^7^ ^]^ For the acquisition of local nano‐IR spectra, the AFM tip was fixed at specific locations, and the nano‐IR spectra were collected in the range of 750–2100 cm^−1^ with a nominal spectral resolution of 8 cm^−1^. The uniquely broad spectrum of synchrotron IR radiation enables full coverage of the desired spectral range and allows the collection of a complete spectrum in a single acquisition. The typical acquisition time of a nano‐IR spectrum was ≈ 12 min for pure PBS buffer through SiC and SiN membranes (data shown in Figure 1B) as well as the fibroblast cells experiments (data shown in Figure 7) and 1 h for DNA/BSA water solution experiments (data shown in Figure 6).

The spectra collected for PBS buffer through SiC and SiN membranes were referenced against a clean Si wafer put in the vicinity of the membrane. The nano‐IR spectra collected from the cells were referenced against the area of the same sample, where no cells were found attached to the membrane, i.e., against the buffer surrounding the cells. The nano‐IR DNA and BSA water solutions spectra were referenced against a pure water spectrum, one single SiC membrane was used to collect spectra of DNA, BSA and pure H_2_O.

The conversion of the recorded interferograms to the infrared amplitude and phase spectra is done using a script developed in‐house at the IRIS beamline in SciLab open‐source software using an asymmetric apodization window based on the three‐term Blackmann‐Harris function and with a zero‐filling factor of four. The nano‐IR spectra represent the reference‐corrected optical phase signal. A linear baseline was subtracted, correction with two anchor points was applied to the obtained optical phase spectra.

The pre‐processing of the s‐SNOM images (topography, mechanical phase, optical amplitude signals) was done in an open‐source software for SPM data analysis (Gwyddion).^[^ ^59^ ^]^ No additional treatment was used for the images shown in Figure 4A,B to avoid mis‐interpretation of the data and erratic calculation of the signal‐to‐noise ratio. For the topography data shown in Figure 5A–C the alignment of the rows (using “median” algorithm implemented in Gwyddion) and the plane correction were done using the area of the rigid frame, excluding the membrane region.

For both the imaging and the spectroscopy modes the detector signal is demodulated at the second harmonic of the AFM cantilever oscillation frequency and the corresponding optical amplitude and phase signals are used to obtain the images and spectra shown. In the experiment on the AFM‐probe – sample mechanical interaction, the 1st and the 3rd harmonics of the optical amplitude signal are also imaged. Use of the heterodyne detection mode and the demodulation harmonics ≥2 allows to isolate the near‐field tip‐sample interaction signal.^[^ ^7^ ^]^ In addition to the optical images, the setup allows to simultaneously collect the AFM images (topography, mechanical phase, and amplitude signals). All measurements are performed at room temperature, having the s‐SNOM continuously purged with dry nitrogen.

### Complementary Optical Microscopy and Infrared Spectroscopy Measurements

Bright field transmission images of the cells were captured using a confocal optical infrared microscope (Hyperion 3000, Bruker Optik GmbH, Ettlingen, Germany) using a pair of 15x Schwarzschild objectives (N.A. 0.4). Bright field reflection images were captured using the optical reflection microscope built in into the s‐SNOM setup (neaScope, Attocube, Haar, Germany) equipped with a 20x long working distance objective (N.A. 0.42, Plan Apo, Mitutoyo, Kanagawa, Japan). The infrared transmission spectra of the membranes were collected with an FTIR microscope (Nicolet iN10, Thermo Fisher Scientific, US) using a pair of 15x Schwarzschild objective (N.A. 0.7) and an internal Globar infrared source. Infrared transmission measurement of a dry cell was done with a confocal optical infrared microscope (Hyperion 3000) coupled to an FTIR spectrometer (Vertex 80) using a pair of 32x Schwarzschild objectives (N.A. 0.52), with 10 × 10 µm^2^ aperture, using the synchrotron infrared radiation, all the components of the spectrometer and the microscope system are from Bruker Optik GmbH, Ettlingen, Germany.

## Conflict of Interest

The authors declare no conflict of interest.

## Author Contributions

The manuscript was written through contributions of all authors. All authors have given approval to the final version of the manuscript.

## Supporting information



Supporting Information

## Data Availability

The data that support the findings of this study are available from the corresponding author upon reasonable request.
