# Supplementary material for: Nano‐Infrared Imaging and Spectroscopy of Animal Cells in Liquid Environment
Source: Small. 2025 Oct 14;21(47):e07097. doi: 10.1002/smll.202507097 (PMC12658920; doi:10.1002/smll.202507097)
Supplement: Supplementary file 1 — Supporting Information [file SMLL-21-e07097-s001.pdf]

NANO · MICRO
small

Supporting Information

for *Small*, DOI 10.1002/smll.202507097

Nano-Infrared Imaging and Spectroscopy of Animal Cells in Liquid Environment

Alexander Veber, Cecilia Spedalieri and Janina Kneipp**

Supporting information for:

Nano-Infrared Imaging and Spectroscopy of Animal Cells in Liquid Environment

Alexander Veber[‡#], Cecilia Spedalieri[‡], Janina Kneipp[‡*]*

[‡] Department of Chemistry, Humboldt-Universität zu Berlin, Brook-Taylor-Str. 2, 12489 Berlin, Germany

[#]Institute for Electronic Structure Dynamics, Helmholtz-Zentrum Berlin für Materialien und Energie GmbH, Albert-Einstein-Str. 15, 12489 Berlin, Germany

E-Mail: Alexander.Veber@helmholtz-berlin.de; Janina.Kneipp@hu-berlin.de

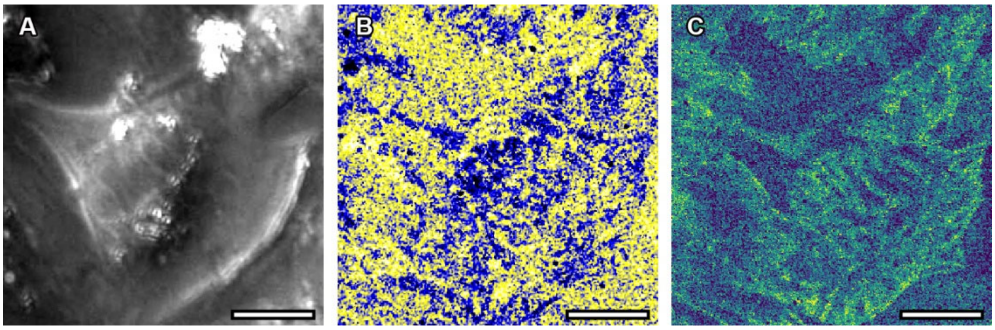

Figure S1. Images of a fibroblast cell in PBS buffer collected using a 30 nm SiC membrane: **(A)** bright field transmission, **(B)** bright field reflection (false-color), **(C)** s-SNOM optical amplitude (s_2). Scale bar is 20 µm

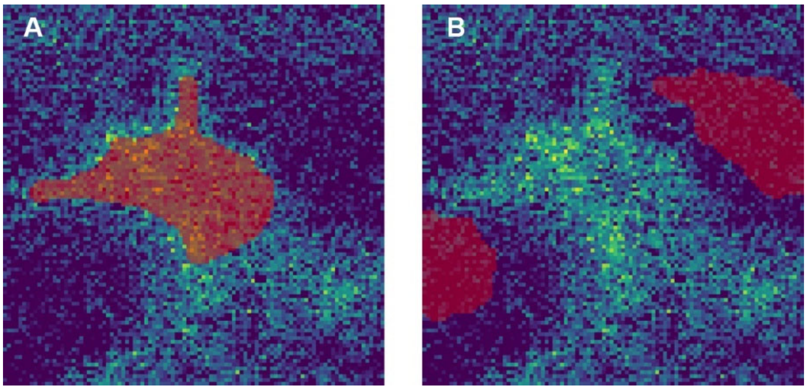

Figure S2. Masks used to calculate signal to noise ratio in the tip-sample interaction optimization experiment: **(A)** mask of the signal area, **(B)** mask of the noise area. The area of the images is 10 x 10 μm^2.

Near-field IR spectroscopy of DNA and BSA water solutions

The spectra of DNA and the protein bovine serum albumin (BSA) molecules in aqueous solutions, shown in Figure 6 of the main manuscript text, were collected through the 30 nm SiC membranes using the IR s-SNOM technique. Exactly the same membrane was used for the measurement of the molecules as well as for acquisition of a pure water spectrum. The latter was used to reference the raw phase shift.

The spectra contain many details and look very comparable to the ones collected via far-field FTIR spectroscopy techniques.[1] The DNA spectrum shows the characteristic bands of phosphodiester groups at approximately 1225 cm^{-1} (antisymmetric) and 1080 (symmetric), the latter with a shoulder at 1063 cm^{-1} assigned to C-O stretching of deoxyribose. Bands related to the stretching of C=O and C=N groups that are involved in base pairing for double stranded DNA are observed in the 1600-1750 cm^{-1} region,[2] for example the adenine-thymine pair at 1695 cm^{-1} and the cytosine-guanine pair at 1650 cm^{-1}. Additional bands can be assigned to the different nucleotides as well as deoxyribose.

The BSA spectrum shows the characteristic amide I, amide II, and amide III bands in the spectral range of 1600-1700 cm^{-1}, 1490-1600 cm^{-1} and 1200-1340 cm^{-1}, respectively, as well as additional spectral features at frequencies below 1200 cm^{-1}. The bands at approximately 1630 and 1655 cm^{-1} in the amide I region can be assigned to the protein secondary structure combination of short unstructured chains[3,4] and α-helix conformations, respectively.[5] The presence of α-helical structure is further confirmed by spectral features at approximately 1545 and 1515 cm^{-1},[6] the latter also having a contribution from a tyrosine ring vibration[4]. BSA has a high content of α-helix in its structure, and the observation of these bands suggest that the protein remains in its native form. It has been shown that when interacting with surfaces like silica, BSA can undergo structural changes decreasing the α-helix content.[7]

Table S1. Tentative assignment of the vibrational bands observed in the nano-IR spectra of DNA and Bovine Serum Albumin water solutions measured through an 30nm SiC membrane. The assignment is based on the data reported in references.[1-7]

DNA	
Wavenumber (cm^{-1})	Assignment
1063	C-O stretching of deoxyribose
1080	PO_2^- groups symmetric
1225	PO_2^- groups antisymmetric
1330	thymine
1377	adenine, guanine
1427	deoxyribose
1451	adenine/deoxyribose
1481	adenine, guanine
1650	cytosine-guanine pair
1695	adenine-thymine pair

BSA	
Wavenumber (cm^{-1})	Assignment
1200-1340	Amide III
1515	Amide II, α-helix conformations and tyrosine ring vibration
1545	Amide II, α-helix conformations
1630	Amide I, short unstructured chains
1655	Amide I, α-helix conformations

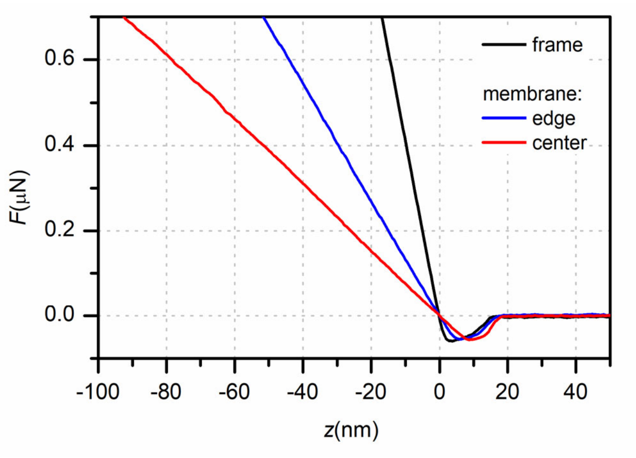

Figure S3. Contact-mode retract curves collected from a 30-nm thick SiC on membrane holder frame(black) area, and the membrane regions close to the edge of the frame (blue) and close to the center (red). The nominal spring constant of 42 N/m was used to convert the as received traces in the force-distance curves.

Conversion of the AFM tapping amplitude and set point into tip–sample distance

In addition to the oscillation amplitude tapping-mode retract curves shown in Figure 5 we analyzed the static deflection data collected simulteneously (Figure S4A). The three static deflection curves collected in tapping AFM mode from different parts of the membrane match well, and for simplicity, we consider the observed signal to represent only the static deflection of the cantilever.

The as-received static deflection data in millivolt (mV) scale were converted to nanometer (nm) scale using the calibration coefficient obtained from the contact mode retract curve measured at the rigid frame (raw data of the trace shown in Figure S3). In this manner we analyzed the static deflection data collected across a range of the set points (data shown in Figure S4A) and different tapping amplitudes (the data corresponding to the oscillation amplitude traces in Figure S4B, the actual data not shown). We found that at a given set-point the static deflection is proportional to the tapping amplitude (A_T) with the maximum value of approximately 7.5 nm at the A_T=130 nm, and negligible at A_T=30nm.

After analyzing a set of oscillation amplitude retract traces collected using AFM tapping mode (Figure 5A and Figure S4B), we determined the equilibrium z-scanner positions corresponding to various settings of the oscillation amplitude and set point. The piezo motor positions were corrected for the static cantilever deflection to obtain the equilibrium tip–sample distance at different cantilever oscillation parameters (Figure S4C).

Assuming the tip oscillates symmetrically around its equilibrium position, the minimum tip–sample distance can be estimated by subtracting half of the in-contact oscillation amplitude. Using the estimated AFM tip-sample distances at different oscillation amplitude and set-point values, we converted the data shown in Figures 4C and 4D into a single plot of the optical signal-to-noise ratio versus minimum tip–sample distance (Figure S4D). As it is seen from the Figure S4D both the tapping amplitude and the set point parameters of the cantilever oscillation enable the control over the sample indentation. Presented in this way, the data demonstrate that signal enhancement can be primarily attributed to the reduced tip–sample distance.

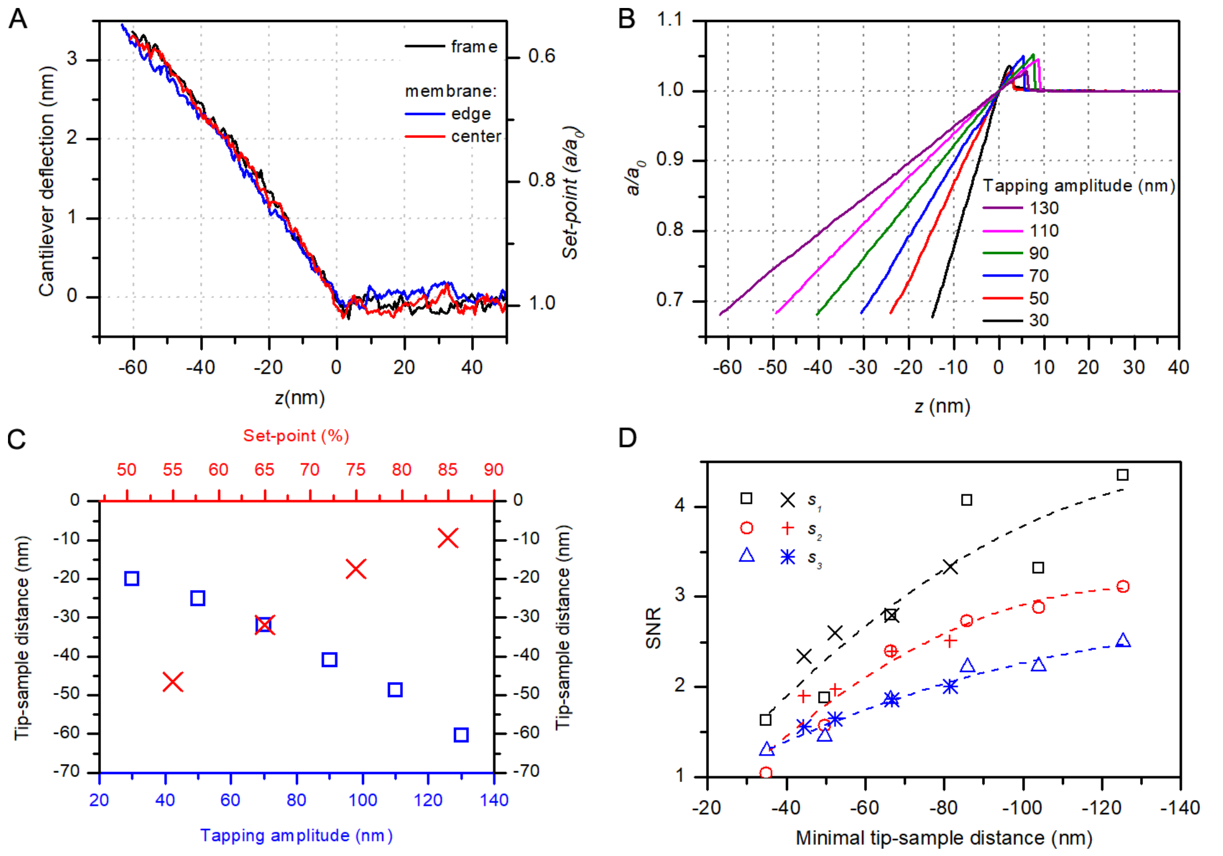

Figure S4. A) Static channel tapping-mode retract curves collected from a 30-nm thick SiC on membrane holder frame(black) area, and the membrane regions close to the edge of the frame (blue) and close to the center (red). The data were acquired simultaneously with the tapping-mode retract curves shown in Figure 5. The data shown in this plot and in the contact-mode data shown in Figure S3 were acquired with the same AFM-probe, which allowed to convert raw mV – scale to nm-scale. B) Retract curves of the normalized tapping amplitude, a/a_0 collected at different tapping amplitude values and set-point of 65% from the center of the 30-nm SiC membrane. C) Estimated equilibrium tip–sample distance at different cantilever oscillation parameters: blue squares represent the data for different tapping amplitudes at the constant set-point of 65%; red crosses show the data for different set point values at the constant tapping amplitude of 70 nm. D) Signal to noise ratio (SNR) of the optical amplitude signal acquired at different demodulation harmonics versus the minimal tip-sample distance. The SNR values are taken from the data shown in Figures 4C and 4D, whereas the tapping amplitude and the set-point abscissae values were converted to the AFM tip-sample distance, as described in the text. Square, circle and triangle symbols represent data from Figure 4C (SNR vs tapping amplitude) and plus-sign, cross and star symbols represent data from Figure 4D (SNR vs set-point) for the 1st, 2nd and 3rd demodulation harmonics of the optical amplitude, respectively. The lines are guides to the eye only.

Comparison of far-field absorption and near-field nano-IR spectra of fibroblasts

In addition to the nano-IR experiment we collected an FTIR spectrum of a dry 3T3 fibroblast cell using diffraction limited IR microscopy using transmission technique (micro-IR). This allows to compare the variation in the spectra between the far-field and near-field methods. The

spectra share several similarities, specifically with respect to the protein for nano-IR and micro-IR method the positions of the Amide I and Amide II band differ only slightly, namely the Amide I band is observed @ 1652 and 1655 cm^{-1} for nano-FTIR and ATR method, respectively (the positions of the band using different techniques is noted hereinafter as 1652/1655 cm^{-1}) and Amide II (1533/1541 cm^{-1}). Other common bands in both nano-IR and micro-IR spectra could be assigned to DNA stretching modes of phosphodiesters (1075/1061-1086 cm^{-1} symmetric, 1213/1236 cm^{-1} antisymmetric), the C-O stretching of carbohydrates (1130/1124 cm^{-1}) and the CH_2 deformation of carbohydrates and lipids (1456/1456 cm^{-1}). At the same time bands in the 1050-1450 cm^{-1} region are better defined in the nano-FTIR spectrum, in particular the bands at 1386 cm^{-1} (CH_3 bending), at 1293 cm^{-1} and 1325 cm^{-1} (amide III) are clearly visible in the nano-FTIR spectrum and hard to identify in the micro-IR measurement. Also, the 1738 cm^{-1} assigned to C=O stretching of lipids is only observed via nano-FTIR technique. The tentative assignment of the observed vibrational bands is given in Table 1 in the manuscript.

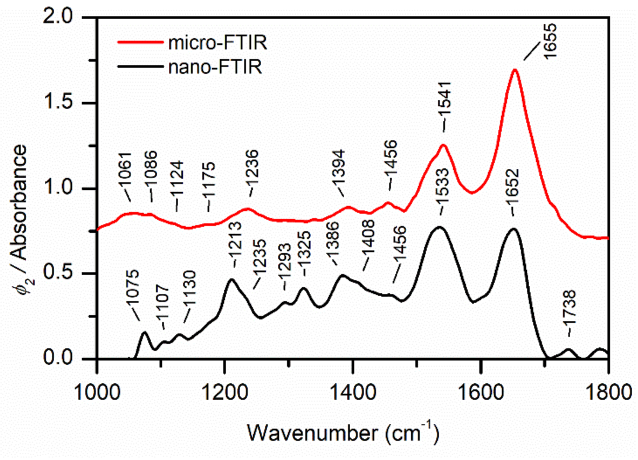

Figure S5. Absorbance micro-IR spectrum of a dry fibroblast cell (red/top trace) and the average nano-IR spectrum (black/bottom trace) calculated using the individual spectra collected for a fixated cell in PBS buffer through SiC membrane (see manuscript text Figure 7A).

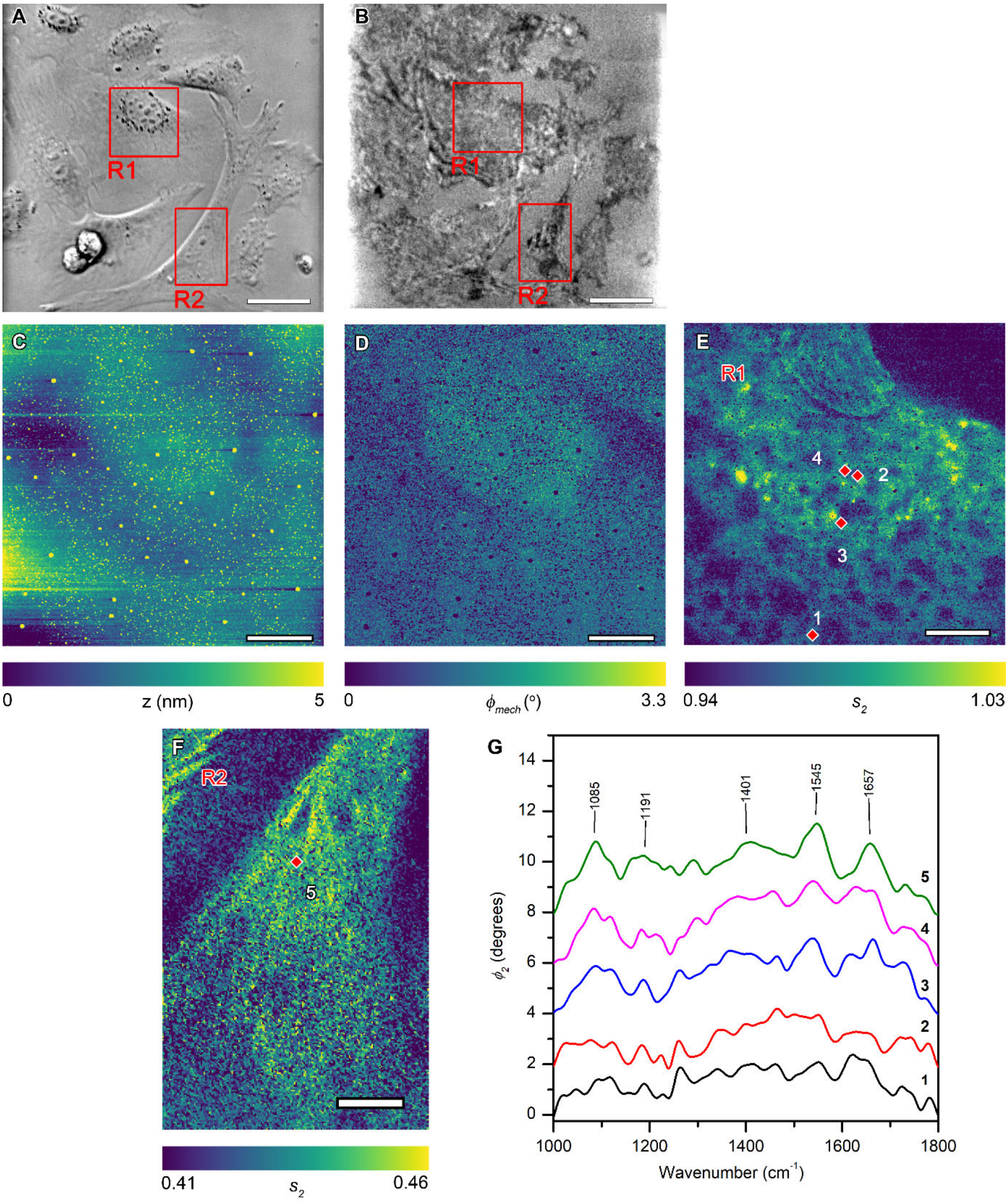

Figure S6. Images and spectra of the fibroblast cells in PBS buffer collected using a 10 nm SiN membrane: **(A)** optical microscope transmission and **(B)** reflection images; **(C)** topology and **(D)** mechanical phase images. **(E)** near-field optical amplitude image over the area R1, **(F)** near-field optical amplitude image over the area R2, **(G)** nano-IR spectra collected at different points of interest, the numbering corresponds to the points marked in panels (E) and (F). The R1 and R2 region are indicated in panels (A) and (B). Scale bar in panels A, B is 50 µm in panels C-F it is 10 µm.

References

(1) Infrared Spectroscopy of Biomolecules, 1st ed.; Mantsch, H. H., Chapman, D., Eds.; Wiley-Liss: New York, NY, 1996.
(2) Wood, B. R. The Importance of Hydration and DNA Conformation in Interpreting Infrared Spectra of Cells and Tissues. Chem. Soc. Rev. **2016**, 45 (7), 1980–1998. https://doi.org/10.1039/C5CS00511F.
(3) Byler, D. M.; Susi, H. Examination of the Secondary Structure of Proteins by Deconvolved FTIR Spectra. Biopolymers **1986**, 25 (3), 469–487. https://doi.org/10.1002/bip.360250307.
(4) Murayama, K.; Tomida, M. Heat-Induced Secondary Structure and Conformation Change of Bovine Serum Albumin Investigated by Fourier Transform Infrared Spectroscopy. Biochemistry **2004**, 43 (36), 11526–11532. https://doi.org/10.1021/bi0489154.
(5) Lenk, T. J.; Ratner, B. D.; Gendreau, R. M.; Chittur, K. K. IR Spectral Changes of Bovine Serum Albumin upon Surface Adsorption. J. Biomed. Mater. Res. **1989**, 23 (6), 549–569. https://doi.org/10.1002/jbm.820230603.
(6) Miyazawa, T.; Blout, E. R. The Infrared Spectra of Polypeptides in Various Conformations: Amide I and II Bands[1]. J. Am. Chem. Soc. **1961**, 83 (3), 712–719. https://doi.org/10.1021/ja01464a042.
(7) Jiang, T.; Hu, X.-F.; Guan, Y.-F.; Chen, J.-J.; Yu, H.-Q. Molecular Insights into Complexation between Protein and Silica: Spectroscopic and Simulation Investigations. Water Res. **2023**, 246, 120681. https://doi.org/10.1016/j.watres.2023.120681.
